# High-Salt Diet Exacerbates *H. pylori* Infection and Increases Gastric Cancer Risks

**DOI:** 10.3390/jpm13091325

**Published:** 2023-08-28

**Authors:** Vyshnavy Balendra, Chiara Amoroso, Barbara Galassi, Josephine Esposto, Claudia Bareggi, Jennie Luu, Lucia Scaramella, Michele Ghidini

**Affiliations:** 1 Saint James School of Medicine, Park Ridge, IL 60068, USA; veeb023@gmail.com; 2 Gastroenterology and Endoscopy Unit, Fondazione IRCCS Cà Granda, Ospedale Maggiore Policlinico, 20122 Milan, Italy; amorosochiara@gmail.com (C.A.); lucia.scaramella@policlinico.mi.it (L.S.); 3 Oncology Unit, Fondazione IRCCS Cà Granda Ospedale Maggiore Policlinico, 20122 Milan, Italy; barbara.galassi@policlinico.mi.it (B.G.); claudia.bareggi@policlinico.mi.it (C.B.); 4 Department of Environmental and Life Sciences, Trent University, Peterborough, ON K9L0G2, Canada; josephineesposto@trentu.ca; 5 The University of the Incarnate Word School of Osteopathic Medicine, San Antonio, TX 78235, USA; jennie.hong.luu@gmail.com

**Keywords:** gastric cancer, *H. pylori* infection, high-salt diet, gastric epithelium, gastritis, mutations

## Abstract

Gastric cancer ranks as the fifth-leading contributor to global cancer incidence and the fourth-highest in terms of cancer-related mortality. Helicobacter pylori (*H. pylori*) infection leads to inflammation and ulceration, atrophic and chronic gastritis, and eventually, increases the risk of developing gastric adenocarcinoma. In this paper, we delve into the combined impact of a high-salt diet (HSD) and concurrent *H. pylori* infection, which act as predisposing factors for gastric malignancy. A multitude of mechanisms come into play, fostering the development of gastric adenocarcinoma due to the synergy between an HSD and *H. pylori* colonization. These encompass the disruption of mucosal barriers, cellular integrity, modulation of *H. pylori* gene expression, oxidative stress induction, and provocation of inflammatory responses. On the whole, gastric cancer patients were reported to have a higher median sodium intake with respect to healthy controls. *H. pylori* infection constitutes an additional risk factor, with a particular impact on the population with the highest daily sodium intake. Consequently, drawing from epidemiological discoveries, substantial evidence suggests that diminishing salt intake and employing antibacterial therapeutics could potentially lower the susceptibility to gastric cancer among individuals.

## 1. Introduction

In 2020, approximately 1.1 million new cases of gastric cancer were diagnosed and 770,000 deaths were associated with it [1]. By 2040, this number is expected to rise to 1.8 million new cases and 1.3 million deaths [2]. The highest incidences were found in males in comparison to females, and 75% of the cases were reported in Eastern Asia (Japan, China, and South Korea) [3,4]. Some common risk factors include *Helicobacter pylori (H. pylori)* infections, specific dietary foods, smoking, and obesity [5].

### 1.1. Genetics

*H. pylori* is a spiral shaped, Gram-negative bacteria that infects and colonizes the stomach of half of the world’s population. This bacterial infection leads to inflammation and ulceration [6]. Individuals infected with *H. pylori* may be asymptomatic, but if left untreated, the bacteria can cause peptic ulcers, as well as atrophic and chronic gastritis. Those with persistent infections have a higher risk of developing gastric adenocarcinoma [7]. The genetic variability of H. pylori is 50-times more diverse than the human population [8]. This is due to the exponential rates of DNA point mutations, causing high genotypic and phenotypic variability in *H. pylori* and intergenomic recombinations [9]. These errors in DNA replication permit *H. pylori* to adapt and thrive in various hosts and changing gastric acidic environments. These DNA replication errors are due to the shortage of DNA repair genes, influenced by the mutagenic properties of DNA polymerase [10]. High genetic diversity allows *H. pylori* to easily incorporate exogenous DNA and transfer their genes to the host gastric cells [11]. Recombinations occurring between genes of the same *H. pylori* strain or between various alleles of *H. pylori* DNA are the driving force behind the genetic variability [12]. The pathogenesis of *H. pylori* is related to specific characteristics of the bacteria, such as the genetic composition of the outer membrane proteins babA1, oipA, and sabA. Genes such as vacA, cagA, iceA, and babA2 have been studied and shown to increase the severity of bacterial pathogenesis, contributing to gastric adenocarcinoma [13]. 

All strains of *H. pylori* have the vacuolating cytotoxin gene (vacA) [14]. Yet, variations specifically in the signal (S) and mid- (M) sections within VacA account for varying cytotoxicities [15]. Differences in the “S” region alter the level of vacuolating activity of VacA, while variations found in the “m” regions signify the specificity of vacuolation, related to toxin binding to recipient hosts. For instance, S1/M1 is the strain of *H. pylori* with the greatest production of toxin, whereas S2/MS will produce a negligible amount [16]. 

#### 1.1.1. CagA

The CagA gene of the bacteria uses a type IV secretion to translate CagA into the host’s gastric cell. Here, it undergoes EPIYA phosphorylation-dependent or -independent processes in the cytoplasm to control the cell’s proliferation and signal transduction pathways [17]. CagA disrupts tight and adherent junctions, compromising DNA integrity and promoting proinflammatory and mitogenic responses. The CagA gene is a marker of pathogenicity [18].

#### 1.1.2. IceA

The IceA gene is composed of two allelic variants known as iceA1 and iceA2 [19]. IceA1 is expressed when *H. pylori* interacts with human gastric epithelial cells. This interaction increases the risk of peptic ulcer disease.

#### 1.1.3. BabA

Blood group antigen binding adhesin (BabA) is encoded by the babA3 gene, and its binding to adhesin has been associated with gastric cancer [20]. 

Overall, studies have shown that outer membrane proteins are more likely to be influenced by genomic mutations. In vivo experiments have revealed that the diversification of *H. pylori*’s outer membrane aids in immune evasion. Thus, the heterogeneity of proteins interacting with the host favors developing chronic infection [21,22].

### 1.2. Clinical Presentation

Clinical presentations of *H. pylori* infections result from virulent strains of the bacteria, molecular host characteristics, lifestyles, and environmental factors [23]. A high-salt diet (HSD) is evidenced as one of the environmental factors increasing the risk of gastric cancer, presenting as the third-most-common cause of cancer deaths globally [24,25]. The American Heart Association suggests that adults should consume less than 2300 mg a day, while the ideal limit of consumption for adults should be no more than 1500 mg [26]. This includes elderly persons who are not taking nutritional supplements or pharmaceutical drugs. Since more than half of the salt intake is from ultra-processed and fast foods, active precautions and preventive measures must be carried out by consumers of these products [27].

**Figure 1:**
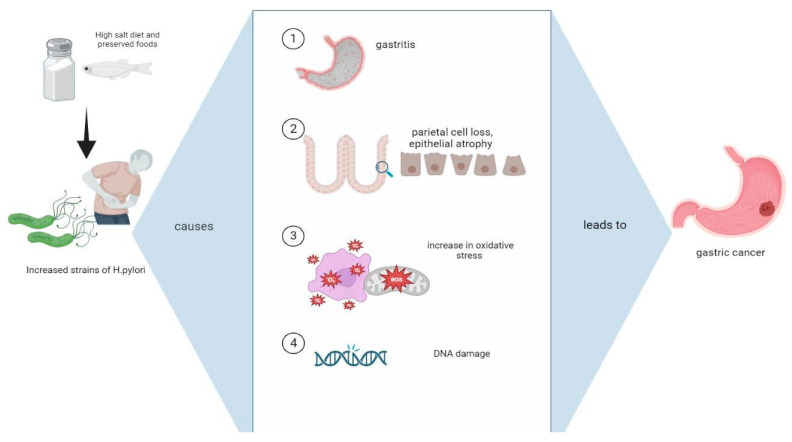
The intake of an HSD results in increased *H. pylori* colonization in gastric mucosa. The increased virulent strains of *H. pylori* lead to: (1) gastritis and ulceration; (2) changes in the structure and function of epithelial cells; (3) high levels of oxidative stress and the production of free radicals; (4) DNA damage. All of these molecular mechanisms increase the risk of developing cancer.

**Figure 2:**
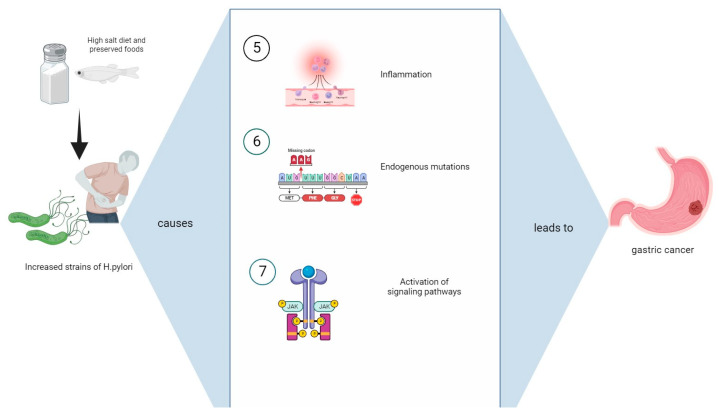
The intake of an HSD results in increased H. pylori colonization in gastric mucosa. The increased virulent strains of H. pylori lead to: (5) inflammation; (6) endogenous mutations; (7) activation of signaling pathways such as JAK/STAT. All of these molecular mechanisms increase the risk of developing cancer.

Many epidemiological studies highlight the synergistic interplay between an HSD and *H. pylori* infections and the increased likelihood of the pathogenesis of gastric cancer [28]. This review aims to synthetize those scientific findings and provide evidence regarding the molecular and genetic mechanisms such as gastric epithelial changes and dysfunction, inflammation, oxidative stress, and the increase in mutations as a result of these risk factors (Figure 1 and Figure 2). Thus, the evolution of *H. pylori* to gastric cancer is heavily influenced by dietary composition, particularly increased salt consumption.

## 2. Materials and Methods

The authors conducted an electronic search across the PubMed, Medline, Cochrane ScienceDirect, Google Scholar, and Embase library databases for English, peer-reviewed articles and reviews published after the year 1993 using the following MeSH terms: gastric cancer, high-salt diet, salt intake, *H. pylori* infection, gastric adenocarcinoma, AND models. Case reports were excluded. The results were further screened by title and abstract for studies performed in rodents and humans, at which time, full-text articles were screened for eligibility.

## 3. Mechanisms Causing Gastric Cancer from the Synergistic Influence of a High-Salt Diet with *H. pylori* Infection

Several mechanisms are outlined whereby an HSD and *H. pylori* synergistically may increase gastric cancer risk, as seen in Figure 1 and Figure 2.

### 3.1. Damage to Mucosal Barrier and Intestinal Metaplasia

Studies indicate that, the parts of the world where increased dietary salt is consumed (preserved fish, soy sauce, pickled foods) and those individuals who are habitual consumers of an HSD parallel the incidence of gastric adenocarcinoma [29,30]. For example, in Asia, where gastric cancer is high, a dietary factor that underlies this cancer risk is the high intake of salt [3,31]. In studies with non-human primates, smoked, pickled, and cured foods revealed a connection between foods with nitrosamines and *H. pylori.* An HSD plays a role in the differentiation of mucosal epithelial cells, as evidenced in intestinal metaplasia and intraepithelial neoplasia in gastric epithelial cells [32]. 

In animal studies, an HSD fostered the colonization of *H. pylori* by altering the structure and stability of the gastric mucosa through an inflammatory response and gastric cell proliferation [33] (Table 1). This has been shown to lead to gastritis, increased epithelial damage, and gastric cancer. Experimental mice who were given NaCl at high concentrations developed gastric mucosal changes with tissue damage and cell proliferation, leading to gastric cancer [34]. *H. pylori*-infected mice were characterized by a loss of parietal cells and hypertrophy of the mucous epithelium in the corpus mucus, compared to the uninfected rodents [35]. Importantly, those *H. pylori*-infected mice showed an exacerbation of inflammation with a high salt intake [36]. In vivo experiments conducted by DeKoster et al. showed that gastric mucosal cells multiplied exponentially with salt intake, a phenomenon similarly seen in *H. pylori*-infected patients [37]. In another study, Fox et al. examined the interaction between an HSD and infection, finding that both promoted gastric injury and advanced gastric lesions to malignancy in the gastric corpus due to hypergastrinemia [33]. Their findings concluded that an HSD may cause carcinogenesis by enhancing *H. pylori* colonization, intensifying chronic *H. pylori* gastritis. 

Additionally, an HSD increased the risk of intestinal metaplasia—a known precursor to gastric cancer [38]. Mucosal damage in the stomach as a result of high intragastric salt concentration coupled with injury from the colonization of *H. pylori* infection increases the rate of mitotic cell division and results in a high rate of cell turnover and the hyperplasia of the gastric epithelium [39] (Table 1). Vencez-Mejez et al. experimented with high-salt injections in rats and noticed pathological indications of irreversible injury in the stomach mucosa—lipid peroxidation, organelle swelling, mitochondrial dysfunction, and fragmented DNA [40] (Table 1). Those animals receiving 12%, 18%, and 24% sodium chloride diets experienced chronic gastritis, regenerative hyperplasia, and focal metaplasia, respectively. In addition, calciform cells were detected in the glands and in gastric foveolar cells, accelerating the development of gastric cancer in the rats.

### 3.2. Foods High in Nitrites

Nitrates, nitrites, and nitrosamines are normally contained in the standard diet. Nitrates and nitrites, naturally contained in fruit and vegetables, are often added to processed meat to allow better preservation, preventing microbial spoilage and maintaining appearance and flavor. Nitrosamines, including N-nitrosodimethylamine (NDMA) as one of the most-common, arise from the chemical reaction from the previous two compounds mentioned [41,42,43].

Nitrates appear to be inert prior to undergoing a reduction–oxidation (redox) reaction caused by mouth bacteria, thus transforming into nitrites. Upon reaching the gastric acid environment, they are converted into nitrous acid, which reacts with amines to form nitrosamines [44]. The presence of nitro-derivatives in the diet leads to the formation of carcinogenic N-nitroso compounds (NOCs) in the GI tract. It has been found that the coexistence of high heme-iron concentrations, typical of a diet high in red and processed meats, enhances these processes [45] by augmenting the carcinogenic risk of these compounds. NOCs contribute to the formation of DNA adducts and are considered as risk factors of gastric cancer, especially non-cardia gastric cancer [46]. A meta-analysis conducted by Song et al. [47] investigated the role of nitro-derivatives on gastric carcinogenesis and found that food rich in nitrates was related to a decreased risk of gastric cancer, while a high intake of nitrites and NDMA resulted in an elevated risk of cancer. The protective role of nitrates was attributed to the fact that dietary nitrates are mainly provided by vegetables rich in fiber, vitamin C, and other antioxidants. However, the authors concluded that, due to the limitations and confounding factors in the studies analyzed, the reliability of these findings was hindered. Another meta-analysis conducted by Zhang et al. [48] included a wide range of participant characteristics and confirmed that high or moderate nitrite intake increases the risk of gastric cancer, whereas a high or moderate nitrate intake was somewhat protective.

*H. pylori* infections have a synergic mechanism with nitro-derivatives, inducing nitric oxide synthase (NOS) in gastric mucosa and neutrophils. Its activity results in an increase in local NO, which, reacting with O2 metals and epithelial-derived hydrogen peroxide (H2O2), can create DNA oxidative adducts. This oxidative stress activates oncogenes and inactivates tumor suppressor genes via hypermethylation of CpG island promoter genes and increased activity of DNA methyltransferase. In addition, NO augmenting DNA damage reduces its repair mechanism by 8-oxoguanine glycosylase [49,50].

Moreover, some studies suggest that gastric atrophy and hypochlorhydria or achlorhydria due to a chronic *H. pylori* infection can increase gastric pH and modify nitrite metabolism, shifting to the production of N-nitrosamine by bacterial nitrite reductases, rather than S-nitrosothiol, a relatively stable NO donor, which has beneficial effects on cardiovascular and metabolic disorders, allowing for a high incidence of gastric cancer development [51,52]. Processed food frequently contains both nitro-derivatives and salt, obtaining a synergic action to promote gastric carcinogenesis [53]. High-dietary-salt intake can change the mucous viscosity protecting the non-cardia parts of the stomach, potentiating exposure to NOCs and leading to cell death. Furthermore, in *H. pylori*-infected gerbils, Toyoda et al. found that a high-salt diet significantly up-regulated the expression of enzymes, among which was iNOS, suggesting an additional pathway for toxicity enhancement in the corpus of the stomach [19,54,55]. 

### 3.3. Alterations in Composition of H. pylori Strains

Between the numerous mechanisms by which a high-salt diet might enhance gastric cancer risk, high-salt conditions in the stomach may have direct effects on *H. pylori*, affecting the expression of different types of genes. It is known that *H. pylori* is endowed with different mechanisms of carcinogenesis, which vary between different *H. pylori* strains [56,57]. One of them is the promotion of gastric epithelial proliferation, oncogenes, and DNA damage via cytotoxin associated gene A (CagA), vacuolating toxin A (VacA), and outer membrane proteins (OMPs) such as blood group antigen binding adhesin (BabA) and sialic acid-binding adhesin (SabA) [51]. Loh et al. [58] used RNA-seq technology to define the salt-responsive transcriptome of *H. pylori* and showed that an increased salt concentration affected the expression of many genes involved in bacterial growth and motility. An increase in the transcription of VacA and OMPs such as sabA, hopA, and hopQ was observed, as well as a reduction in the transcription of fecA2 and fecA3 (specialized OMPs involved in iron transport), suggesting an adjustment of *H. pylori* growth and a reduced motility in response to high-salt stress conditions. Caston and colleagues analyzed the *H. pylori* exoproteome cultured with different concentrations of sodium chloride. High-salt concentrations within the extracellular environment increased the levels of VacA in the extracellular space. Two possible mechanisms were proposed: the first, and more certain, was un-upregulated VacA transcription, while the proposed second process was an increased VacA release from the outer membrane into the extracellular space [32]. The decrease in motility, which may favor *H. pylori* attachment to gastric cells, might provide *H. pylori* the capacity to adapt both in the short term and the long term to a high-salt environment [59]. 

Previous studies [60,61] showed strain-specific differences in the effect of salt on CagA expression or survival in response to salt stress, while additional studies confirmed that *H. pylori* strains with gene mutations involved in iron metabolism had a positive selection both in vivo [59,62] and in vitro high-salt conditions [63]. This highlights the possible crucial role played by specific selected strains to determine the *H. pylori*-induced disease outcome. Furthermore, additional studies in animal models described a positive interaction between a high-salt diet and *H. pylori* infections with an upregulation of processes involved in oxidative stress (COX-2 and iNOS), inflammatory response, *H. pylori* colonization, and damage progression [19,55,64]. These findings may strengthen the hypothesis of a correlation between high-salt diets and increased stomach cancer risk, driven by the change of interactions of the bacteria with host cells.

### 3.4. Oxidative Stress

At equilibrium, reactive oxygen species (ROSs) play a role in cellular function and metabolism and assist in both the innate and adaptive defense mechanisms [65]. This contributes to the overall maintenance of the cell. The perturbation between ROSs and the antioxidant system is known as oxidative stress, with an overproduction of free oxygen radicals [66], potentially resulting in molecular dysfunction. Studies have shown that an HSD negatively impacts the gastric immune system [67]. Tamura et al. showed salt to be an aggressive factor, creating gastric mucosal lesions. A highly osmotic pressure environment induced by salt inhibits the mitochondrial electron transfer system, producing superoxide anions. These anions permit apoptotic cell death via caspase-3 activation and gastric injury via denervation in epithelial cells [68].

Decreased function in immunity due to increased oxidative stress caused by sodium overload allows *H. pylori* to easily infect the gastric epithelial cells and promote tumorigenesis [69,70]. *H. pylori* produces immense quantities of superoxide anions to combat the nitric oxide made by host inflammatory cells, leading to gastric epithelial cell damage. Once inside the host cell, *H. pylori* virulence factors further increase oxidative stress through the activation of signaling pathways [71]. *H. pylori* strains containing CagA and VacA are able to invade and destroy gastric epithelial cells and stimulate cell proliferation towards dysplasia [72,73]. Processes that activate the immune and antioxidant systems of the host organism are inhibited once *H. pylori* inhibits gastric cells [74]. Specifically, CagA *H. pylori* strains continue to contribute to the toxic environment of free radical ions, through the expression of spermine oxidase (SMO), and give rise to hydrogen peroxide. Hydrogen peroxide is a powerful oxidizing agent, which further contributes to the production of free radicals, creating hydroxyl radicals. SMO is able to induce caspase-mediated apoptosis and contributes to DNA strand breakage, furthering the maintenance of the low-energy state [75,76] (Table 1). In addition, the CagA strain gives rise to tumor necrosis factor-alpha (TNFα), which contributes to an already continuous cycle of inflammation and oxidative stress [77], while interleukin (IL)-8 carries pro-oncogenic properties [78] (Table 1). On the other hand, vacuolating cytotoxin A (VacA) adds to ROS production by activating nuclear factor kappa B (NFκB) through Ca2+ influx [14]. VacA inhibits autophagy in gastric epithelial cells while creating an environment prompting cancer pathogenesis [79,80] (Table 1). Lastly, another virulence factor known as γ-glutamyltransferase in the bacteria releases many radical ions, further damaging gastric epithelial cells [81]. In combination, the oxidative stress emitted from a hypertonic environment, as well as that induced by *H. pylori* cause oxidative DNA damage and lead to the progression of inflammation to carcinoma.

### 3.5. Endogenous Mutations

A chronic *H. pylori* colonization of the human stomach has been correlated with an increased risk of gastric adenocarcinoma development [82]. Moreover, *H. pylori* has been classified as a class I carcinogen [83]. Despite the high genetic diversity among *H. pylori* isolates, the *H. pylori* cag pathogenicity island (cag PAI) is a strain-specific virulence factor. This factor augments cancer risk by encoding a type IV secretion system that facilitates the delivery of an oncoprotein, CagA, into the cytoplasm of gastric epithelial cells [84]. Once inside gastric epithelial cells, the CagA protein interferes with multiple intracellular signaling pathways and confers to the host cells cancer-hallmark capabilities.

CagA, once tyrosine-phosphorylated, is capable of binding to SHP2, a non-receptor typeof PTPase, characterized by being enzymatically inactive under normal physiological conditions. However, once the binding of the CagA protein to SHP2 occurs, permanent activation of PTPase occurs, leading to downstream activation of MEK-ERK kinases, a pro-oncogenic signaling pathway involved in the proliferation and differentiation of cells [85]. The sustained proliferative signaling profile of CagA is also linked to its ability to physically interact with the E-cadherin, an adherence junctional component functionally associated with Wnt signaling, as well as epithelial–mesenchymal transition (EMT) [86]. The CagA/E–cadherin interaction affects the E-cadherin/β-catenin complex’s formation. The membranous fraction of β-catenin translocates to the nucleus, where it activates the Wnt target genes, including certain transcription factors (CDX1 and CDX2) that activate the stemness-associated reprogramming factors Sal-like protein 4 (SALL4) and Krüppel-like factor 5 (KLF5) [87].

CagA also hampers the p53 pathway in several ways. Among them, CagA promotes p53 proteasomal degradation by activating E3 ubiquitin ligases, human double-minute (2HDM2), and ARF-binding protein 1 (ARF-BP1) [88]. CagA also contributes to spontaneous loss-of-function mutations in the p53 gene. Specifically, cagA-positive *H. pylori* in the gastric epithelium promotes the expression of a DNA/RNA editing enzyme (AID) responsible for nucleotide alterations, resulting in spontaneous loss-of-function mutations in the TP53 tumor suppressor gene [89]. Furthermore, although the molecular mechanism has not been assessed yet, it has been demonstrated that chronic exposure to the CagA protein in gastric epithelial cells induces PAR1b inhibition, leading to defects in microtubule-based mitotic spindle formation and double-strand breaks [90]. Remarkably, *H. pylori* cagA-positive-induced gastritis is associated with CpG hypermethylation of MGMT, the gene encoding the DNA repair protein O6-methylguanine DNA methyltransferase (MGMT) [91]. All this evidence indicates that CagA in host gastric epithelial cells may play a key role in the development of intestinal metaplasia.

**Table 1:** Mechanisms leading to gastric cancer from the concurrent influence of a high-salt diet with *H. pylori* infection.

*Inflammation* Effects of an HSD with *H. pylori* Infection	Mechanisms Leading to Gastric Cancer	References
Mucosal barrier damage	Increased mitotic cell division and turnover	[32,33,37,38,39,40]
	Induction of intestinal hyperplasia and metaplasia	[38,39]
Cellular damage	Irreversible lipid peroxidation, organelle swelling, and mitochondrial dysfunction	[40]
Alteration of *H. pylori* gene expression	Increased expression of CagA, VacA, BabA, and SabA	[14,32,50,58,59,79,80,84,85,86,87,88,89,90,91]
Oxidative stress via ROSs	Apoptosis of gastric cells via the production of superoxide anions and hydrogen peroxide	[68,71,75,76]
Inflammation	Activation of TNFα, interleukins, COX-2, and Th17	[19,55,64,77,78,92,93,94,95,96,97,98]

Legend: *H. pylori*: Helicobacter pylori, HSD: high-salt diet, ROSs: reactive oxygen species, TNFα: tumor necrosis factor-alpha, COX-2: cyclooxygenase 2.

The relationship between *H. pylori* and inflammation is complex and multifactorial. It is known that *H. pylori* infection is the main cause of chronic inflammation in the stomach [92], which stimulates an inflammatory response intended to eliminate the bacteria (Table 1). However, an exacerbated immune response damages the stomach lining, leading to chronic inflammation [93]. *H. pylori*-induced gastroenteritis contributes to mucosal injury by inducing both humoral and cellular immune responses. It has been demonstrated that *H. pylori*-induced inflammation is characterized by the activation of inflammatory cells and the release of cytokines, such as interleukin (IL)-1β, IL-6, and tumor necrosis factor (TNF)-α. These cytokines promote the recruitment and activation of additional immune cells, further exacerbating the inflammatory response [94]. Prolonged inflammation leads to histological changes, including pre-neoplastic gastric lesions, and generates reactive radicals that might disrupt the host’s DNA [95]. The release of IL-1β, IL-6, IL-8, IL-17, and TNF-α mobilizes tumor-associated neutrophils involved in metastases by inducing epithelial–mesenchymal transition (EMT) through the activation of the JAK2/STAT3 and ERK1/2 signaling pathways in gastric cancer cells [96] (Table 1).

*H. pylori* also triggers a Th17 response, which is associated with a lower survival and increased metastasis. IL-17 can activate proinflammatory pathways such as NFκB and c-Jun N-terminal kinase (JNK), and high levels of JNK have been found in several cancer cell lines [97]. Moreover, IL-17 can also attract neutrophil migration conferring pro-tumorigenic functions, as described in colorectal cancer lesions [98].

As a note, *H. pylori* is able to conceal itself from the immune system by modifying the surface molecules to avoid the recognition from the toll-like and pattern recognition receptors (TLRs and PRRs) of dendritic cells [99]. Moreover, when *H. pylori* pathogen-associated molecular patterns (PAMPs) interact with PRRs, adaptive cellular mechanisms such as oxidative stress and autophagy are activated, turning on several transcriptional pathways including MAPK, NFκB, Wnt/β-catenin, and PI3K. These pathways are associated with (i) the production of inflammatory cytokines, (ii) changes in cell proliferation and differentiation, (iii) the activation of angiogenesis, (iv) EMT, and (v) immunological tolerance [100].

### 3.6. Management of Helicobacter Pylori Infection: Antibiotics, Probiotics, Natural Treatments, and Future Perspectives

*H. pylori* infection treatment consists of a combination of three or four drugs, such as combinations of acid suppressants with antibiotics and/or bismuth. The first-line treatment should be decided according to locoregional or individual *H. pylori* antibiotic resistance. The second-line treatment should consider the drugs used in the first-line treatment and the antibiotic resistance status as well [101]. Antibiotics used as the first-line treatment are clarithromycin, amoxicillin, metronidazole, levofloxacin, and furazolidone. Acid suppression is obtained from proton-pump inhibitors (PPIs) such as omeprazole, esomeprazole, lansoprazole, pantoprazole, or rabeprazole at a double-standard dose. The increase of intragastric pH to a value of 6 or higher is essential in order to optimize the stability, bioavailability, and efficacy of antibiotics [102]. PPI efficacy is increased by doubling the PPI standard dose and should be considered in the case of first-line treatment failure [103]. Antibiotic resistance is the main reason for treatment failure, especially in the case of PPI-based triple-therapies (PPI-TTs), which include a PPI, clarithromycin, and amoxicillin or metronidazole as a substitution for either amoxicillin or clarithromycin [104]. Clarithromycin resistance has increased to 15–30% worldwide, while metronidazole resistance is >25% in most areas of the world. However, while the evaluation of antibiotic susceptibility testing (AST) is crucial for clarithromycin use, in vitro AST for metronidazole does not correlate with clinical efficacy. Moreover, metronidazole has synergistic efficacy together with co-administered drugs, particularly bismuth [101].

Clarithromycin-based PPI-TT is the treatment of choice in the case of local clarithromycin resistance prevalence <15%. On the contrary, in cases of higher resistance rates, the treatment of choice is a bismuth-based quadruple-therapy composed of a PPI, bismuth, tetracycline, and a nitroimidazole antibiotic (BiQT) [105]. BiQT has a high eradication efficacy of 90%, which does not require AST. It is also not affected by clarithromycin resistance and overcomes metronidazole resistance with the use of bismuth [106]. Another possible first-line treatment in cases of high rates of clarithromycin resistance is the concomitant therapy (PPI plus three antibiotics simultaneously administered) [107]. Concomitant therapy has been found to be superior to PPI-TT and may be an alternative to BiQT in case of the unavailability of the quadruple-therapy. Table 2 reports the possible first-line treatments available [108].

In case of the failure of the BiQT treatment, the second-line treatment consists of a levofloxacin-based regimen [109]. However, levofloxacin has a high resistance rate (up to 20% in Europe and 18% in the Asia-Pacific region), and because of this, its use is limited even in second-line regimens [110]. Levofloxacin-based regimens include amoxicillin and a PPI. In cases of higher resistance rates, alternative choices are PPI + rifabutin + amoxicillin (or clarithromycin in case of a penicillin allergy). A possible choice may also be high-dose PPI + amoxicillin [102]. Adverse events to treatment occur in 30–70% of patients and include nausea, diarrhea, and taste disturbances [111]. The prevalence of diarrhea is 1–15% and is caused mainly by alterations of the gut microbiota and overgrowth of opportunistic pathogens [112].

Probiotics have an adjuvant role in the treatment of *H. pylori*, both by improving the eradication rate of *H. pylori* infection and reducing the rate of adverse events given by antibiotic therapy. A network meta-analysis of 34 randomized controlled trials comparing nine types of different probiotic treatment showed that Bifidobacterium–Lactobacillus and Bifidobacterium–Lactobacillus–Saccharomyces performed better than others, achieving satisfactory results both in eradication and side-effect evaluation and improving standard anti-*H. pylori* triple therapy [113]. Other effective probiotics reported to diminish bacterial load are Saccharomyces boulardii and L. johnsonni La1 [114].

Moreover, Lactobacillus reuteri and its antipathogen compound reuterin were found to be effective both in inhibiting *H. pylori* and reducing the side effects of the antibiotic treatment. Reuterin was reported to downregulate the vacA and flaA genes, responsible for *H. pylori* virulence [115].

Among natural treatments, curcumin (diferuloylmethane) and polyphenolic plant metabolites have shown anti-proliferative activity against *H. pylori*. For instance, curcumin interacts with CagA by suppressing its oncogenic activity [116]. Recently, an essential oil mixture, obtained from species from the genera Satureja L., Origanum L., and Thymus L., called HerbELICO^®^, was shown to inhibit the growth of 20 different *H. pylori* types regardless of their resistance or host origin with immediate bactericidal activity and the ability to penetrate through mucin [117]. Many different plant extracts and phytochemicals have been tested for anti-*H. pylori* activity. However, very few of them have been investigated in vivo for efficacy and capability to inhibit *H. pylori* urease activity [118,119]. Additionally, a diet focused on the reduction of salt intake is key to managing *H. pylori* infection [68]. As indicated previously, high sodium levels in the diet can lead to the onset of pre-malignant lesions in the gut and increase the susceptibility of acquiring an *H. pylori* infection. Thus, limiting HSD can help prevent and even manage *H. pylori* infections, as well as potential cancers.

On the whole, *H. pylori* antibiotic resistance demands the development of new antimicrobial drugs and different treatment strategies that are able to increase cure rates and reduce relapses. Inhibition of *H. pylori* urease and blocking of flagellar function are possible targets. Moreover, the development of polymeric nanoparticles delivering antibiotics and other anti-*H. pylori* agents may increase the penetration of drugs into the mucus layers. The development of selective probiotics against gastric pathogenic bacteria may be important, as well. Indeed, *H. pylori* infection alters the biodiversity of other gastric bacteria, with the development of carcinogenic clusters such as Septostreptococcus, Streptococcus, Parvimonas, Prevotella, Rothia, and Granulicatella. Lastly, further research is warranted in order to identify effective vaccine candidates both for preventive and therapeutic purposes [86].

## 4. Discussion

*H. pylori* infection and an HSD are risk factors for gastric cancer. Analyses were completed to discern how these two factors contribute to the progression of gastric cancer. First, high salt intake was shown to alter gastric cells, leading to the hyperplasia of the gastric pit epithelium, resulting in endogenous mutations. Second, metaplastic changes eventually lead to malignant tumors due to damage from a high intragastric salt concentration, which highly aggravates the epithelium, as well as increases metaplastic alteration. Third, carcinogenic N-nitroso compounds are occasionally found in highly salty foods, which are regularly consumed by populations living in East Asia. In the presence of salt, these compounds can induce additional toxicity and, thus, be detrimental to cells. Fourth, *H. pylori* colonization thrives in an HSD. *H. pylori* has many virulence determinants such as cag-positive strains, which become more lethal in the presence of salt and can inhibit gastric epithelial cells. Lastly, the protective barrier, which is composed of a viscous mucus, is destroyed, creating immune instability. This allows the host to become susceptible to the infection, resulting in chronic inflammation. The release of numerous, harmful cytokines to the epithelium leads to gastritis and ulcerations, which are precancerous. Thus, the above-mentioned evidence presents the mechanisms by which a highly concentrated salt environment has a synergistic role with *H. pylori.* These interactions enhance the probability of developing gastric cancer [119,120]. Although it is difficult to quantify how much an HSD could contribute to the risk of gastric cancer development especially in the absence of *H. pylori* colonization, previous case–control studies reported a higher median sodium intake for gastric cancer patients with respect to healthy controls. Moreover, the risk of gastric cancer was significantly increased by *H. pylori* infection, but only in the case of high sodium intake (highest third of daily sodium intake estimated by a food frequency questionnaire). Differently, *H. pylori* infection did not increase gastric cancer risk for other measures of salt exposure [121]. As far as tumor location is concerned, mice fed with an HSD showed increased proliferation in gastric proximal corpus and antrum and a concomitant multifocal reduction in parietal cell number in the proximal corpus. This process resulted in a general elongation of gastric pits, promoting subsequent *H. pylori* colonization and carcinogenesis [33]. In addition, a Chinese-population-based case–control study supported the view that HSD increases the risk of non-cardia gastric cancer [122].

There are several limitations that impact the nature of the review. First, only articles that were published in English were considered, and a search was not performed on the grey literature. Potentially, there could be studies conducted in other languages (especially among the Japanese population), which could equate to the total number of eligible studies being much larger than the number analyzed in this review. In addition, differences in pathogenetic processes may be present among different ethnicities (Western vs. Eastern). Moreover, many of the studies carried a moderate level of bias relating to confounding variables, as most of the research did not accurately adjust for factors such as smoking, obesity, consumption of red meat, and alcohol, which could have impacted the reliability and validity of the results. The synergistic effects of an HSD and *H. pylori* infection on gastric epithelial cells and subsequent damage have only been studied in various mouse models. However, the effects of an HSD should be considered in other animal models to verify the consistency of the outcomes. Importantly, the high concentration of dietary salt was inconsistent across all studies and was not operationally defined when modeling salt intake and gastric cancer risk.

## 5. Conclusions

This review demonstrated that a high-salt diet and *H. pylori* infection could increase the risk of gastric cancer, especially in the East Asian population. This study has important public health implications. Gastric cancer will lead to a serious global cancer burden, and therefore, prevention can be taken through the administration of medical treatments to eradicate H. pylori. Individuals who prefer salt-burdened foods or are habitual consumers of salt need to undergo dietary education, as well as management to reduce its consumption. A review of the aforementioned studies showed urinary sodium/creatinine ratios as a reliable measure of salt intake. Thus, more experiments utilizing this measure of salt intake with larger sample sizes are needed to further confirm these results. Consideration should be given to conducting more randomized controlled trials and cohort studies to assess the effects of increased and reduced dietary salt intake on *H. pylori* and the risk of gastric cancer based on subject differences.

## Figures and Tables

**Table 2:** First-line treatment for *Helicobacter pylori* infection.

Therapy Name	Dosing	Duration (Days)	Eradication (%)
Clarithromycin-based PPI-TT	PPI bid + clarithromycin 500 mg bid + amoxicillin 1 gr bid or metronidazole 500 mg tad	14	70–85
Bismuth-based quadruple	PPI bid + bismuth salicylate 300 mg qid + metronidazole 500 mg tid + tetracycline 500 mg qid	14	75–90
PPI-TTT	PPI bid + amoxicillin 1 gr bid + clarithromycin 500 mg bid + metronidazole 500 mg tid	14	90

Legend: bid: twice a day, PPI: proton pump inhibitor, qid: four times a day, TT: double-antibiotic therapy, TTT: triple-antibiotic therapy, tid: three times a day [108].

## Data Availability

Not applicable.
